# Report on the Occurrence of Anisakid Nematodes in Commercial Fish from the Southwestern Indian Waters

**DOI:** 10.3390/pathogens15101023

**Published:** 2026-09-29

**Authors:** Anitha Antony, Thangaraj Raja Swaminathan, Kua Beng Chu, Masazurah Abdul Rahim, Dewi Syahidah, April Hari Wardhana, Shubhadeep Ghosh, Mona Saleh, Gokhlesh Kumar, Maria Juviann Isaacs, Sekar Gobinath

**Affiliations:** 1 Microbiology, Fermentation and Biotechnology Division, ICAR-Central Institute of Fisheries Technology, CIFT Junction, Willingdon Island, Kochi 682029, India; 2 Department of Biotechnology, Faculty of Sciences, Cochin University of Science and Technology, Kochi 682022, India; 3 Department of Fisheries Malaysia, Fisheries Research Institute, Jalan Batu Maung, Penang 11960, Malaysia; 4 Research Centre for Veterinary Science, National Research and Innovation Agency, Jl. Raya Jakarta-Bogor KM 46, Cibinong 16911, West Java, Indonesia; 5 Fisheries Science Division, Indian Council of Agricultural Research, Krishi Anusandhan Bhawan-II, Pusa, New Delhi 110012, India; 6 Clinical Division of Fish Health, University of Veterinary Medicine, Veterinärplatz 1, 1210 Vienna, Austria; 7 Marine Biotechnology, Fish Nutrition and Health Division, ICAR- Central Marine Fisheries Research Institute, Kochi 682018, India

**Keywords:** *Anisakis typica*, *Auxis thazard*, nematodes, morphological identification, molecular identification

## Abstract

Anisakid nematodes are zoonotic parasites with a heteroxenous life cycle involving diverse hosts across multiple trophic levels in the marine ecosystem. Anisakiasis represents a significant global public health issue, with numerous clinical cases documented across the regions where raw and undercooked fish and fishery products are consumed as delicacies. The presence of *Anisakis* larvae, especially within fish muscle tissue, substantially decreases both the market value and consumer acceptability of fish products. However, their occurrence in Indian marine fishes remains underexplored. Therefore, the present study investigates anisakids in nine commercially important marine fish species sold in the local markets of the southwestern Indian coast through morphological and molecular approaches. A total of 10 anisakid nematodes were recovered from the visceral cavity of frigate tuna, *Auxis thazard*. Nematodes exhibited the characteristic morphology of the third larval stage of *Anisakis* spp. Prevalence, mean intensity and mean abundance of larvae were 16.67%, 2.0, and 0.33, respectively. Phylogenetic analysis suggested that *Anisakis typica* is closely related to isolates from the Asia-Pacific region. Low genetic variation was observed among *A. typica* isolates from the current study and other geographically distinct locations. The study provides a groundwork for expanding our knowledge on the occurrence of anisakids in commercially important fish from the southwestern Indian waters and their potential implications in food safety and public health.

## 1. Introduction

The nematodes of the genus *Anisakis* (Anisakidae family) have an indirect life cycle involving marine mammals, mainly cetaceans, dolphins, and seals, which serve as definitive hosts harbouring the adult stage of the larvae. The adult worms release embryonated eggs that subsequently develop into L1 and L2 stages in the marine environment. The first intermediate host, viz., copepods and small crustaceans, ingests the L2 stage, which advances to the L3 stage. A wide range of fish and cephalopods that prey on these first intermediate hosts act as the paratenic hosts, transporting L3 larvae to the definitive hosts. The fish and cephalopods are also a transmission link to the accidental host, i.e., humans, making them notorious fish-borne zoonotic parasites and etiological agents of anisakiasis, a condition characterised by gastrointestinal and/or allergic manifestations in humans [1]. Anisakiasis is a global public health concern, with its incidence mainly attributed to the consumption of raw or undercooked seafood containing infective third-stage larvae (L3). Multiple clinical cases have been reported primarily across Asia and Europe, where traditional culinary practices involve minimal cooking of the seafood [2]. Besides being an emerging public health hazard, these parasites negatively impact consumer perception and marketability, compromising food safety and quality. This incurs additional costs for quality checks or processing in high-risk regions, subsequently resulting in substantial financial losses to the fisheries sector and seafood processing industries [3].

In India, the existing literature records the occurrence of anisakids. For instance, the genus *Anisakis* was reported from *Clarias batrachus* in Tripura [4], and *Anisakis simplex* was recovered from diamondback squid, *Thysanoteuthis major*, in the southeastern Arabian Sea [5]. However, these reports were based only on morphological features. As the L3 stage infecting the teleost fishes and cephalopods lacks diagnostic morphological characters, the identification relying exclusively on morphology is insufficient for species-level discrimination [6]. A recent report of *Anisakis typica* infection in Indian sand whiting, *Sillago sihama* from the central west coast of India considered both morphological and molecular aspects to identify anisakids [7]. It is evident that information on the paratenic host range remains sparse, despite their vast diversity and commercial significance in India. Moreover, the lack of adequate molecular data on anisakids hinders the understanding of their genetic diversity and zoogeography. The first documented incidence of rare pulmonary anisakiasis in the southern region of India [8] further necessitates detailed investigations on the occurrence of these zoonotic parasites in the Indian context.

The study aims to investigate the presence of anisakids in commercially important marine fishes caught off the Indian coast. It further intends to infer the taxonomic position of these nematodes using morphological and molecular approaches, along with an analysis of the phylogeny and genetic variations.

## 2. Materials and Methods

### 2.1. Collection of Fish

A total of 720 teleost fish specimens, belonging to nine commercially important species, were purchased from local markets around Kochi, Kerala, between November 2024 and April 2025, based on seasonal availability. The specimens represented 9 species: *Rastrelliger kanagurta*, *Sardinella* sp., *Atule* sp., *Nemipterus* sp., *Selar* sp., *Stolephorus* sp., *Sphyraena* sp., *Leiognathus* sp., and *Auxis thazard* (Table 1).

### 2.2. Anisakid Nematodes Examination

Parasitological examination was performed using a stereomicroscope (RSMr-3, Radical Scientific Equipments Pvt., Ltd., Ambala, India). The body cavity, viscera, and gonads of the dissected fish specimens were examined for nematode parasites. The fillets were sliced into thin slivers of 1–2 mm thickness and inspected under visible-light illumination. The nematodes were carefully recovered from the host tissues, washed thoroughly in physiological saline solution, enumerated and stored in 70% ethanol until further analysis. A total of ten nematodes were examined under a light microscope (CILIKA TF-50 Digital, Medprime Technologies Pvt. Ltd., Thane, India) without chemical clearing to identify those exhibiting characteristic features of anisakids. The Field Emission Scanning Electron Microscopy of two larval nematodes was carried out using a MAIA3 Triglav^TM^ (Tescan Group, Brno, Czech Republic) at DST-SAIF following the standard facility protocol. Conventional sample preparation was not performed prior to imaging. The larvae were identified to genus level using existing morphological keys [6,9,10]. Quantitative parasitological descriptors—prevalence, mean intensity and mean abundance—were calculated following Bush et al. [11] using QPweb v. 1.0.15 [12]. Prevalence was expressed as the percentage of fish infected among the total number of fish examined, with 95% confidence interval (CI) estimated using the Clopper–Pearson method. Mean intensity was calculated as the average number of parasites among the infected individuals, while mean abundance was defined as the total count of parasites divided by the total number of both infected and uninfected fish specimens. Estimates of mean intensity and mean abundance were reported with 95% CI determined using bias-corrected and accelerated (BCa) bootstrapping resampling with 2000 iterations.

### 2.3. PCR Amplification

For the molecular analysis, genomic DNA of individual larvae was extracted using the DNeasy^®^ Blood and Tissue kit (Qiagen, Hilden, Germany,) following the manufacturer’s instructions. PCR amplification of the Internal Transcribed Spacer (ITS1-5.8S-ITS2) region of ribosomal DNA was performed according to Kuhn et al. [13] using the primer set: NC2 5′-TTA-GTT-TCT TTT-CCT-CCG-CT-3′ and TK1 (5′-GGC-AAA-AGT-CGT AAC-AAG-GT-3′), with some modifications in the thermocycling conditions. PCR reactions were prepared in a total volume of 50 μL containing 25 μL of 2X EmeraldAmp GT PCR Master-Mix (Takara Bio Inc., Kusatsu, Japan), 3 μL each of 10 μM forward and reverse primers, 14 μL of nuclease-free water, and 5 μL extracted genomic DNA (5 ng/μL), resulting in a final template amount of 25 ng (0.5 ng/μL). The initial denaturation step was carried out at 98 °C for 2 min, followed by 30 cycles of the denaturation step at 98 °C for 15 s, primer annealing at 57 °C for 30 s, and extension at 72 °C for 45 s, with a final extension at 72 °C for 10 min. A negative control, i.e., a reaction without template DNA, was included in the PCR run. The PCR products were analysed by gel electrophoresis and visualised using a gel documentation analysis system (Bio-Rad Laboratories Inc., Hercules, CA, USA). Six representative positive PCR products were sent to GeneSpec Pvt. Ltd., Kochi, India, for Sanger sequencing.

### 2.4. Phylogenetic Analysis

The obtained sequences were compared with those available in the GenBank database using the basic local alignment search tool (BLAST v.2.16.0.) (https://blast.ncbi.nlm.nih.gov/Blast.cgi, accessed on 15 November 2025) for species-level identification [14]. The nucleotide sequences obtained were deposited in GenBank under accession numbers PV892391, PV892393, PV953655–PV953658. Reference sequences and the outgroup *Ascaris lumbricoides* retrieved from the NCBI database (*n* = 38), along with sequences generated in this study (*n* = 6), were used for the construction of a Maximum Likelihood phylogenetic tree based on the Kimura 2-parameter model of nucleotide substitution with 1000 bootstrap replicates in MEGA v.12 software [15,16,17]. The number of haplotypes, polymorphic sites, haplotype diversity (Hd), nucleotide diversity (π), and neutrality tests, including Tajima’s D and Fu’s Fs statistics, was estimated using DNA Sequences Polymorphism software (DnaSP) v.6 to elucidate the genetic variability [18].

## 3. Results

### 3.1. Anisakid Nematodes

**Figure 1:**
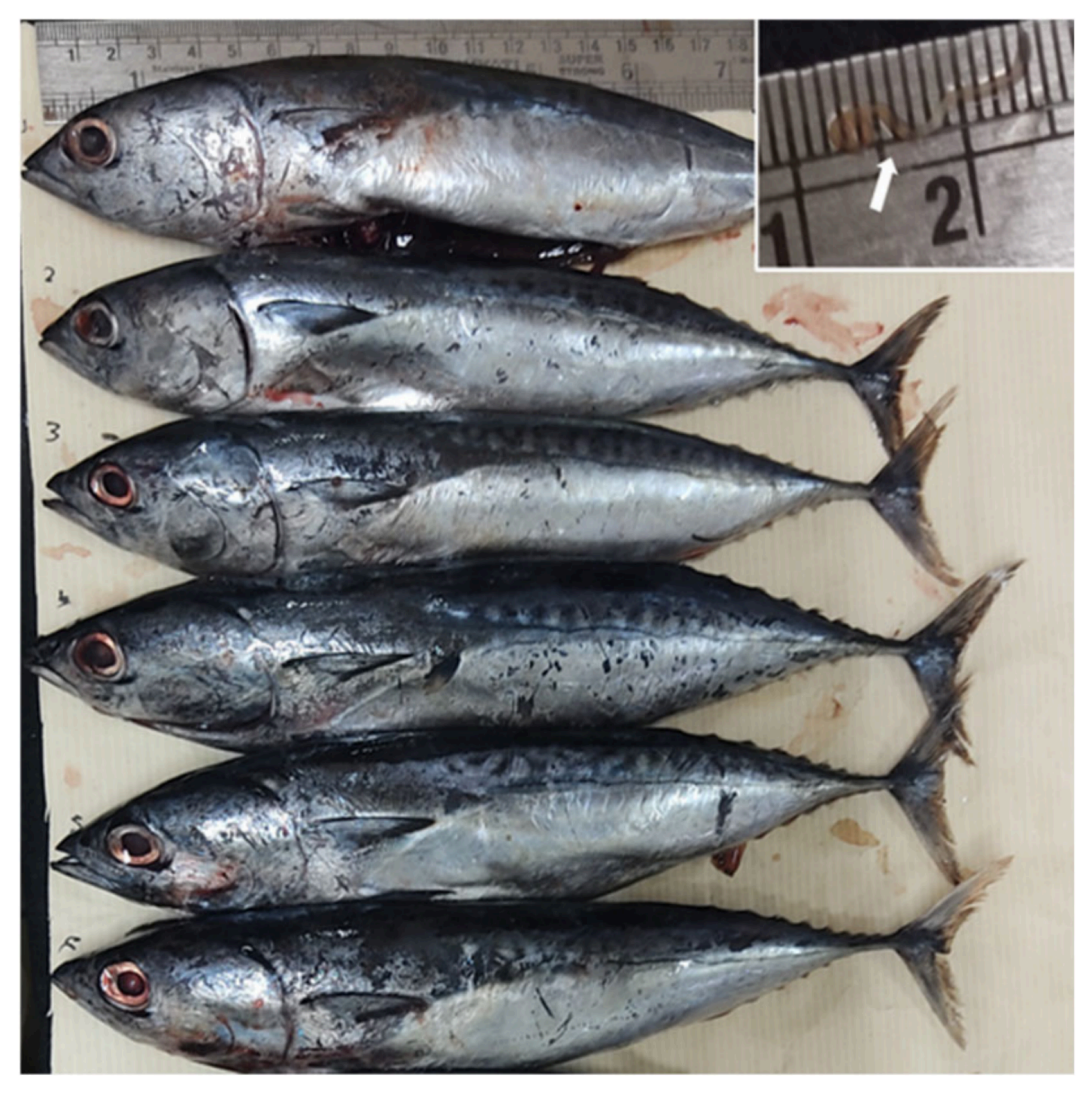
*Auxis thazard* specimens collected from a local market in Kochi for parasitological examination. Inset shows the anisakid nematode (arrow) recovered from the visceral cavity of *A. thazard*.

**Figure 2:**
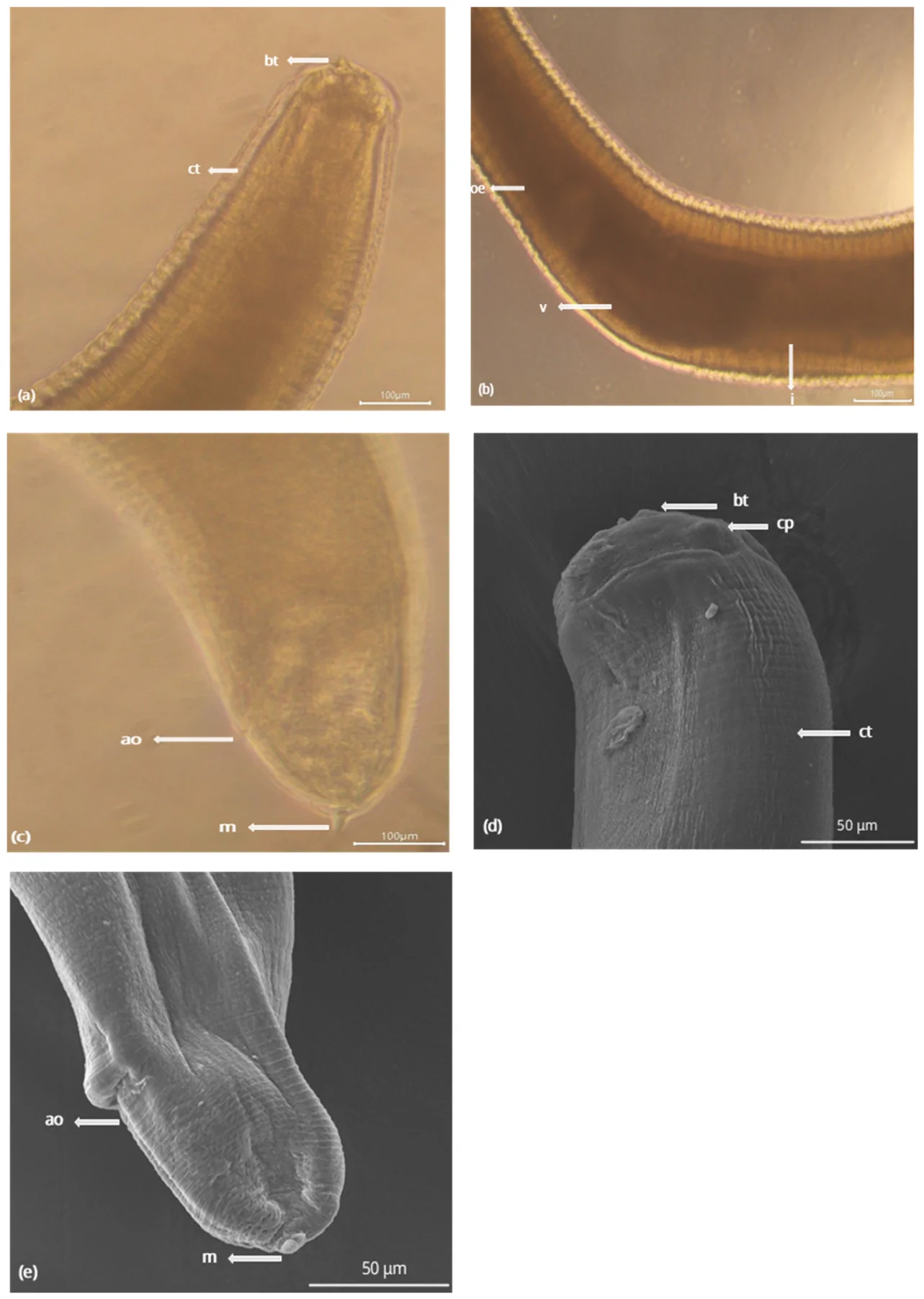
Light micrographs of L3 stage larvae of *Anisakis* spp.: (**a**,**d**) anterior end; (**b**) digestive tract; (**c**,**e**) posterior end. Abbreviations: bt, boring tooth; cp, cephalic papillae; ct, cuticle; oe, oesophagus; v, glandular ventriculus; i, intestine; ao, anal opening; m, mucron.

A total of 10 nematodes possessing characteristic anisakid morphological features were recovered from the visceral cavity of *A. thazard* specimens (Figure 1). Prevalence, mean intensity, and mean abundance were 16.67% (95% CI: 0.056–0.347), 2 (95% CI: 1–3), and 0.33 (95% CI: 0.0872–0.794), respectively. Microscopic examination (Figure 2) revealed that L3 larvae possessed a cylindrical body with a thick cuticular layer with annular transverse striations and were attenuated towards both extremities. A distinct boring tooth and an inconspicuous lip were visible in the cephalic end. A triradiate mouth opening with two dorsolateral and two ventrolateral cephalic papillae was observed in SEM images of the anterior region. The muscular oesophagus was followed by a glandular oesophagus, i.e., ventriculus, which joins the intestine. The anal opening was seen at the posterior extremity, which also bore a short, conical tail with a rounded tip and a spine-like protrusion, i.e., a terminal mucron [6,9,10].

### 3.2. PCR and Phylogenetic Analysis

**Figure 3:**
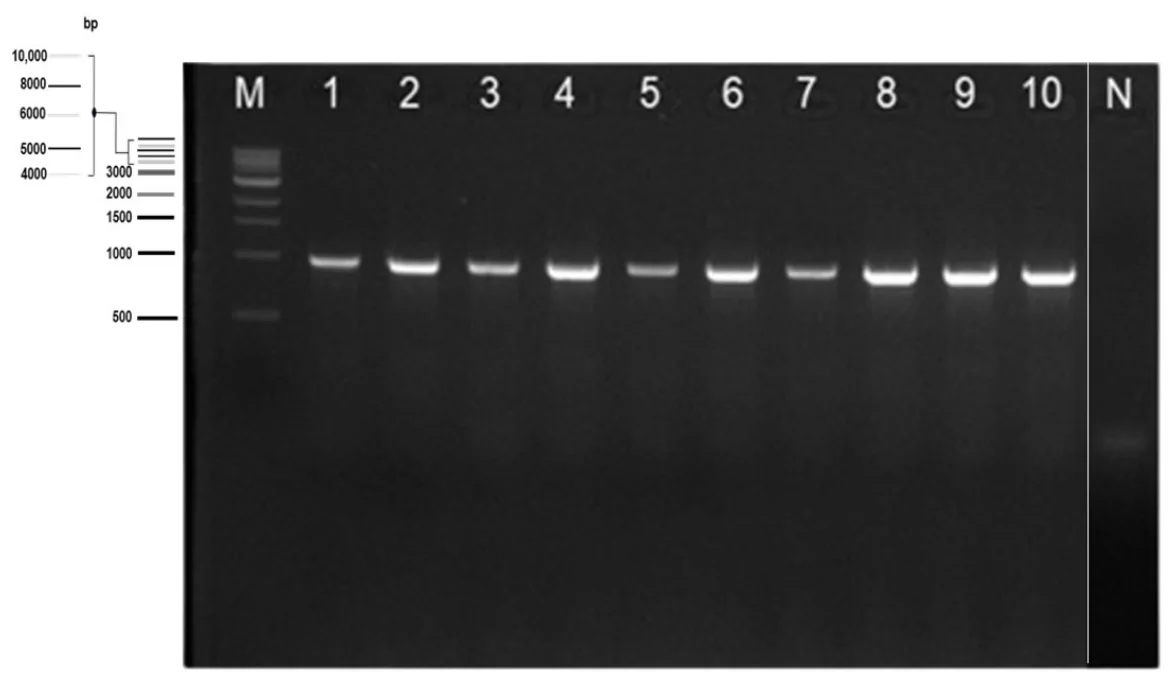
PCR-amplified products of the ITS region of rDNA resolved using agarose gel electrophoresis. M: 1 Kbp DNA ladder; Lane 1–10: PCR-amplified genomic DNA of anisakid nematodes; N: Negative control. The vertical white line indicates the splice.

**Figure 4:**
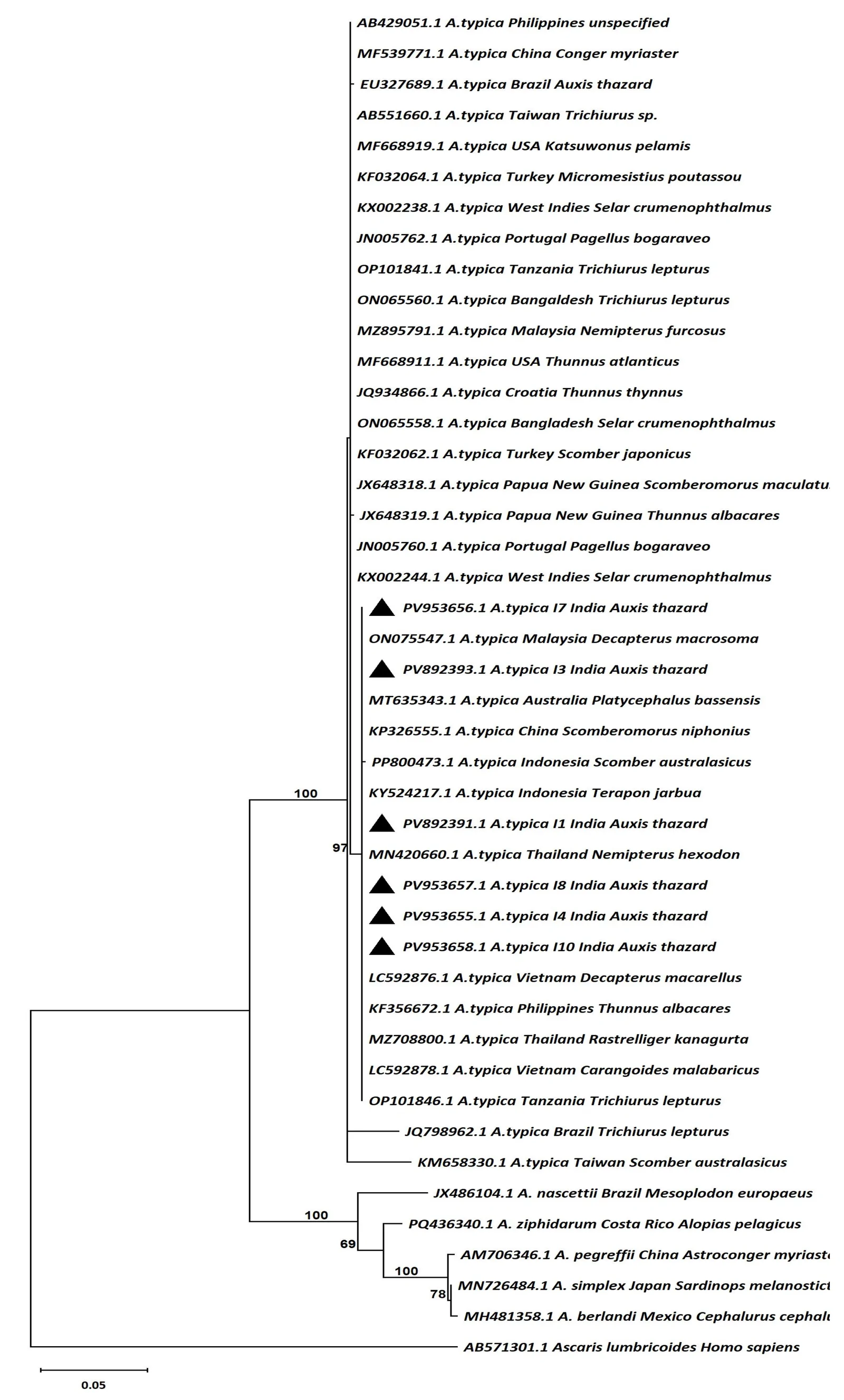
Maximum likelihood tree constructed with 1000 bootstrap replicates based on the ITS region of *Anisakis typica* from India and other references retrieved from GenBank. The numbers at the nodes represent maximum-likelihood bootstrap support values based on 1000 replicates; values < 50% are omitted. The nucleotide sequences obtained in this study are labelled.

ITS primer sets amplified 900 bp of the rDNA region of anisakid nematodes (Figure 3). Sequence homology analysis of the ITS1-5.8S-ITS2 region of rDNA from representative nematodes revealed 100% identity with *A. typica* sequences available in GenBank. The sequences of *A. typica* were distinct from those of other *Anisakis* species. Within the *A. typica* clade, the six sequences from this study clustered with Indo-Pacific isolates from Indonesia, Malaysia, Thailand, the Philippines, China, Vietnam, Australia, and Tanzania; however, several internal branches showed low bootstrap support (<50%) (Figure 4).

Intraspecific genetic variations were elucidated based on the ITS region from the six Indian sequences and 32 *A. typica* sequences from multiple geographic locations. Six haplotypes were identified from the dataset with 26 polymorphic (segregating) sites, including 23 singleton variable sites and 3 parsimony informative sites. Haplotype diversity was estimated to be 0.613 ± 0.045, while nucleotide diversity (pi) was 0.00417. Tajima’s D test showed a significant negative value. Fu’s F-statistic was also negative (−4.33426, *p* < 0.02). No genetic polymorphism was observed among Indian sequences. Haplotype distribution analysis revealed that Indian sequences shared a haplotype with Indonesia, Malaysia, Thailand, the Philippines, China, Vietnam, Tanzania, and Australia. Sequences from the Philippines, Malaysia, Taiwan, China and Tanzania also constituted a separate haplotype along with Bangladesh, Portugal, Turkey, Croatia, the West Indies, and Papua New Guinea. The two sequences from Brazil, along with one sequence each from Papua New Guinea and Taiwan, were present as distinct haplotypes.

## 4. Discussion

Investigations into the occurrence of anisakid nematodes in economically significant fishes in southwestern India using morphological and molecular approaches identified frigate tuna, *A. thazard,* as a paratenic host for *A. typica*. No anisakid infection was detected in the other examined specimens. To the best of our knowledge, this study provides the first record of *A. typica* in *A. thazard* from this region. Furthermore, the recovered *A. typica* exhibited limited intraspecific variation among conspecific isolates from multiple geographic locations.

It is well established that anisakids exploit a spectrum of teleost fish as paratenic hosts, including members of orders Scombriformes, Carangiformes, Clupeiformes, Spariformes and Acanthuriformes [1,19]. The presence of anisakids in *A. thazard* and their absence in the other examined species may be attributed to the ecology of predator–prey populations, such as feeding behaviour, habitat preference, and trophic position [20]. *A. thazard* (Scombridae) is an epipelagic fish distributed across tropical and subtropical waters and is an economically significant wild catch from the southwestern coast of India, with the landings exceeding 6000 tonnes during 2015–2020. *A. thazard* has been previously reported to host trematode, cestode and copepod parasites [21,22].

*A. typica* is widely documented in warm tropical or temperate oceans, particularly in coastal areas of the Atlantic, Pacific and Indian Oceans inhabited by its preferred definitive host, i.e., delphinids [23]. To date, frigate tuna as a paratenic host of *A. typica* has been reported only from the Atlantic Ocean along the coast of Brazil and the Makassar Strait in Indonesia [24,25,26]. Generally, *A. typica* has been reported to occur in the visceral cavity or is found coiled on the visceral organs like the liver, gonads or intestine [27]. Similarly, the nematodes were observed within the body cavity in the current study. The study employed visual inspection for the examination and recovery of anisakids, as it is widely employed in rapid screening for anisakids. Although this technique may underestimate the infection levels due to low efficiency, particularly when the fillets or the slivers are thick [28].

Morphological identification aided by microscopic techniques remains the conventional approach for nematode identification. In the current study, this approach was efficient for identifying the nematode larvae to the genus level. The exclusive reliance on anatomical features for taxonomical classification of anisakids is insufficient, as several diagnostic characters are distinguishable only during later stages of the developmental cycle [3]. In addition, external environmental and ecological factors further complicate accurate discrimination of the taxonomic position, particularly when performed by non-specialists [23]. Thus, molecular techniques, such as sequence analysis, are increasingly performed alongside morphological or macroscopic identification.

Genetic markers, such as the ITS of ribosomal RNA gene, mitochondrial cytochrome oxidase-I and II, metallopeptidase nas10, elongation factor α-1 and microsatellites, are widely employed for species-level discrimination between members of the genus *Anisakis* [29]. The study utilised the ITS region of rDNA for the species-level identification, phylogenetic inference, and assessing the intraspecific genetic variations. *A. typica* analysed in the current study exhibited close associations with sequences from other geographic locations, with pronounced clustering within the Asian region, suggesting minimal variation. These inferences are consistent with sequence analysis of *A. typica* from Thailand and Vietnam [6,10,30]. This observation is also in agreement with the analysis of intraspecific genetic variations. The haplotype diversity of *A. typica* from India and other geographic locations indicates that there is a certain degree of moderate differentiation among the populations. The occurrence of both shared and distinct haplotypes across geographic locations may be due to the wide distribution and migratory nature of the paratenic and definitive hosts, since these hosts contribute to the dispersal of the larvae [31]. Several members of the Delphinidae family, such as *Stenella* sp. and *Steno* sp., with tropical distributions, have been recorded along the coast of Kerala in southwestern India, likely facilitating the dissemination of the anisakid larvae [32].

The dietary preference of *A. thazard* for crustaceans, forage fishes, and cephalopods, along with its distribution across the Indian Ocean driven by monsoon currents, substantiates its role as a paratenic host in transmitting L3 larvae [33,34]. The statistically significant negative values of neutrality tests indicate deviations from neutral evolution due to an excess of rare alleles and low-frequency polymorphisms, which is suggestive of a recent population expansion, although the limited dataset analysed in the current study may limit the robustness of the conclusion [35]. There is a great deal of controversy regarding the zoonotic risk of *A. typica* since the species is mainly recorded from viscera and there is no molecular evidence in clinical cases, which prevents any definite conclusions from being reached. However, documented evidence of migration of *A. typica* to the musculature, along with mouse-model studies demonstrating its ability to induce inflammation, desquamation of the gastric mucosal epithelium and haemorrhage, raises concern regarding the risk of potential human infections [27,36]. Therefore, this work on the occurrence of anisakids from *A. thazard* contributes to expanding current knowledge of the paratenic host range of *A. typica* in Indian waters. Our work provides a groundwork for further assessment of zoonotic and food safety risks associated with nematodes in marine fish species through improved understanding of parasite ecology, invasion dynamics, population structure, and trophic interactions in Indian waters.

Although the current study offers new information regarding the presence of anisakid nematodes in commercial marine fish species, it does have some limitations. Firstly, detection of the anisakids was mainly dependent on visual examination of the fish tissues, and this could have led to an underestimation of both the prevalence and intensity of infection, especially in the case of larvae situated in deeper tissues or muscles. Secondly, the molecular analysis was solely based on the ITS region of rDNA that provides limited resolution for phylogenetic inference. The relatively small number of *A. typica* isolates available for molecular analysis also restricts the interpretation of demographic patterns. The fact that anisakids were not found in the other fish species examined might be due to differences in sample size, infection intensity, seasonal occurrence, and host ecology. Moreover, the present study did not directly evaluate their zoonotic potential or pathogenicity in humans.

## 5. Conclusions

Our study surveyed the occurrence of anisakid nematodes in commercial marine fish species from the southwestern Indian waters. We identified anisakid nematodes from the visceral cavity of frigate tuna, *A. thazard,* by morphological identification and PCR sequencing. ITS ribosomal DNA sequencing identified the nematodes as *A. typica.* Furthermore, phylogenetic analysis showed that *A. typica* is closely related to isolates from the Asia-Pacific region. This study provides basic knowledge on the occurrence of anisakids from marine fish in the southwestern Indian waters, and these data could be used to study the food safety risks associated with nematodes in marine fish species. Further study is required to develop more sensitive and comprehensive nematode detection methods, including artificial digestion and systematic examination of both viscera and edible muscle tissues, to obtain reliable estimates of prevalence, intensity, and tissue distribution. Additional mitochondrial and nuclear markers, along with substantially larger sample sizes, are recommended for a more robust assessment of the genetic and phylogeographic structure of *A. typica*.

## Figures and Tables

**Table 1:** Details of the fish specimens examined for anisakid nematode infections in the present study.

Sl. No.	Examined Species	No. of Fish Specimens Collected	Mean Length ± S.D. (in cm)	No. of Infected Fish
1	*Rastrelliger kanagurta* (Indian mackerel)	150	21.05 ± 1.47	-
2	*Sardinella* sp. (Indian Sardine)	270	15.38 ± 2.50	-
3	*Nemipterus* sp. (Threadfin bream)	120	16.05 ± 4.16	-
4	*Atule* sp. (Yellowtail scad)	30	28.29 ± 0.91	-
5	*Selar* sp. (Bigeye scad)	30	19.75 ± 0.77	-
6	*Stolephorus* sp. (Indian anchovy)	30	9.44 ± 0.44	-
7	*Sphyraena* sp. (Barracuda)	30	22.24 ± 1.12	-
8	*Leiognathus* sp. (Ponyfish)	30	10.49 ± 0.45	-
9	*Auxis thazard* (Frigate tuna)	30	24.44 ± 0.62	5

Note: Sl. No.: serial number; No. of fish specimens collected: number of fish specimens collected; S.D.: standard deviation; No. of infected fish: number of infected fish.

## Data Availability

The nucleotide sequences obtained were deposited in GenBank under the accession numbers PV892391, PV892393, and PV953655–PV953658.

## Abbreviations

The following abbreviations are used in this manuscript:

CI	Confidence Interval
ITS	Internal Transcribed Spacer
rDNA	Ribosomal DNA
